# Review of Aboriginal child health services in remote Western Australia identifies challenges and informs solutions

**DOI:** 10.1186/s12913-019-4605-0

**Published:** 2019-10-26

**Authors:** Philippa J Dossetor, Kathryn Thorburn, June Oscar, Maureen Carter, James Fitzpatrick, Carol Bower, John Boulton, Emily Fitzpatrick, Jane Latimer, Elizabeth J Elliott, Alexandra LC Martiniuk

**Affiliations:** 1 Clinical Medical School, College of Medicine, Biology & Environment, Australian National University, ACT, Canberra, 2605 Australia; 2 Nulungu Research Institute, University of Notre Dame, Broome, Australia; 3 Marninwarntikura Women’s Resource Centre, Crossing, Fitzroy, Australia; 4 Nindilingarri Cultural Health Services, Crossing, Fitzroy, Australia; 5 University of Sydney, Sydney Medical School, Sydney, Australia; 6 Telethon Kids Institute, The University of Western Australia, Perth, Australia; 7 University of Newcastle, Newcastle, Australia; 8 The Sydney Children’s Hospital Network (Westmead), Sydney, Australia; 9 The George Institute for Global Health, Sydney, Australia; 10 The University of Toronto, Toronto, Canada

**Keywords:** Indigenous, Remote Australia, Child health, Health services, Coordination, Integration, Fetal alcohol Spectrum disorders

## Abstract

**Background:**

Despite a national focus on closing the gap between Aboriginal and non-Aboriginal child health outcomes in Australia, there remain significant challenges, including provision of health services in very remote communities. We aimed to identify and map child health services in the very remote Fitzroy Valley, West Kimberley, and document barriers to effective service delivery.

**Methods:**

Identification and review of all regional child health services and staffing in 2013. Verification of data by interview with senior managers and staff of key providers in the Western Australian Country Health Service, Kimberley Population Health Unit, Nindilingarri Cultural Health Services and non-government providers.

**Results:**

We identified no document providing a comprehensive overview of child health services in the Fitzroy Valley. There were inadequate numbers of health professionals, facilities and accommodation; high staff turnover; and limited capacity and experience of local health professionals. Funding and administrative arrangements were complex and services poorly coordinated and sometimes duplicated. The large geographic area, distances, extreme climate and lack of public and private transport challenge service delivery. The need to attend to acute illness acts to deprioritise crucial primary and preventative health care and capacity for dealing with chronic, complex disorders. Some services lack cultural safety and there is a critical shortage of Aboriginal Health Workers (AHW).

**Conclusions:**

Services are fragmented and variable and would benefit from a coordinated approach between government, community-controlled agencies, health and education sectors. A unifying model of care with emphasis on capacity-building in Aboriginal community members and training and support for AHW and other health professionals is required but must be developed in consultation with communities. Innovative diagnostic and care models are needed to address these challenges, which are applicable to many remote Australian settings outside the Fitzroy Valley, as well as other countries globally. Our results will inform future health service planning and strategies to attract and retain health professionals to work in these demanding settings. A prospective audit of child health services is now needed to inform improved planning of child health services with a focus on identifying service gaps and training needs and better coordinating existing services to improve efficiency and potentially also efficacy.

## Background

*An 8-year old child living 150 km from the very remote service town is seen in the paediatric clinic in Fitzroy Crossing and suspected to have fetal alcohol spectrum disorder (FASD). Problems with learning and academic achievement, oppositional behaviour, attention and hyperactivity, writing and growth are identified. Current skin infections (scabies and impetigo), suppurative otitis media, severe dental caries and asthma are also diagnosed. The child requires assessment by the school psychologist and ongoing management by paediatric, allied health, community health, dental, and child and adolescent mental health (CAMH) services.*

The disparity in child health and developmental outcomes between Aboriginal and non-Aboriginal children living in remote Australian communities is well described [1, 2]. Although few population-based studies are available [1, 3] data consistently demonstrate a high burden of health needs and demand for services for Aboriginal children. One retrospective cohort study of Aboriginal infants in remote Northern Territory communities reports high rates of hospital admission and visits to remote primary health centres beginning in early childhood and notes the inadequacy of services to meet demand [3].

**Fig. 1:**
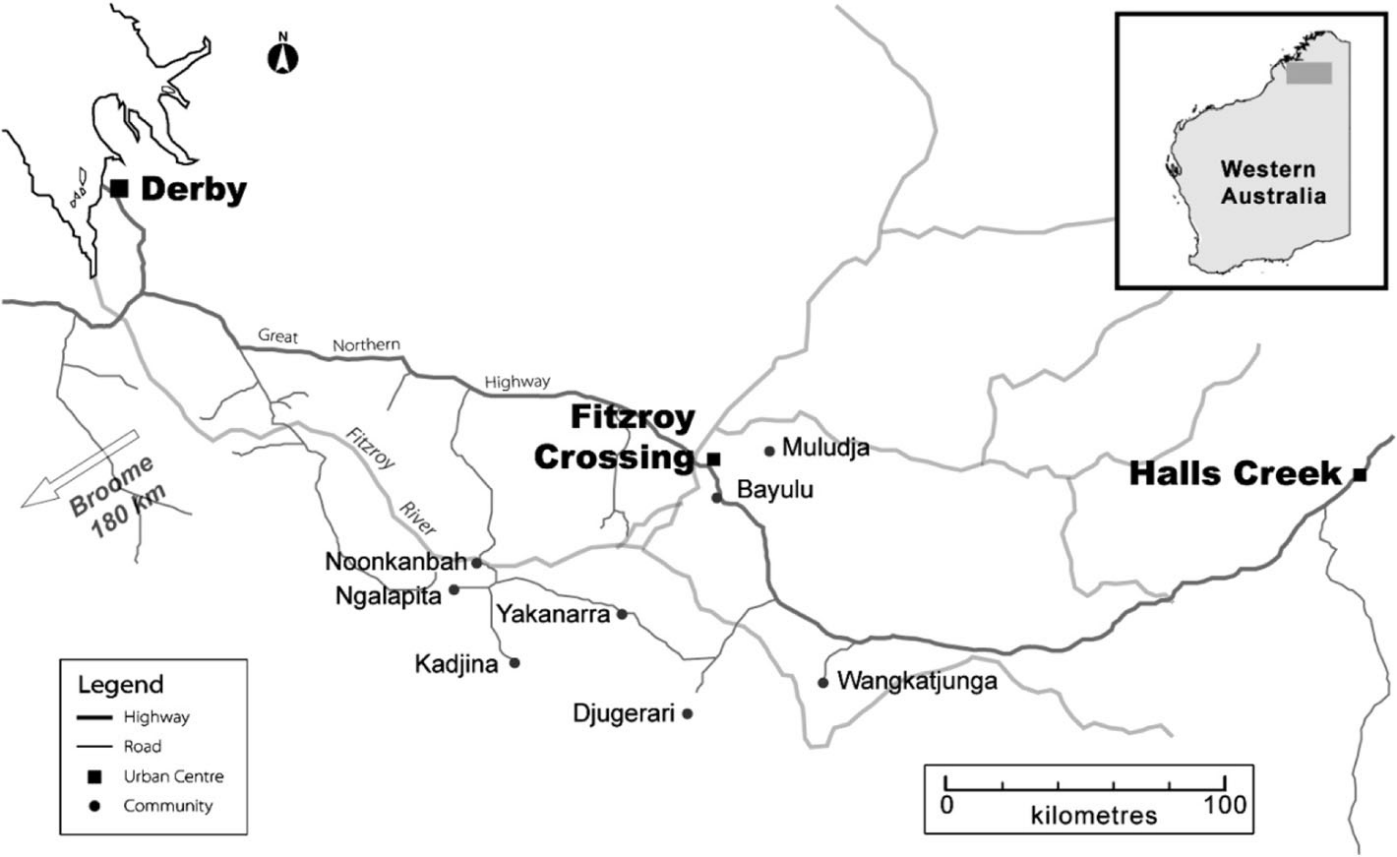
Fitzroy Crossing and surrounding communities

Population-based data from the Lililwan Project in the Kimberley’s Fitzroy Valley in Western Australia suggest a similar scenario [4]. The Lililwan Project was initiated by Aboriginal communities to determine the prevalence of Fetal Alcohol Spectrum Disorder (FASD) and health and developmental problems [4–6]. The Fitzroy Valley (Fig. 1) incorporates 45 communities serviced by the town of Fitzroy Crossing, all of which are classified as very remote by the Accessibility/Remoteness Index of Australia [4, 7]. Fitzroy Crossing has a population of approximately 1600, including about 1000 Aboriginal people. Surrounding communities account for another 1500, mainly Aboriginal, people [8, 9]. According to Communicare™, a patient database used in the Kimberley, there were approximately 1400 children under the age of 16 living in the Fitzroy Valley in 2013 [9, 10].

Since 2009, assessment data on Australia’s five-year-olds in the Australian Early Development Census (AEDC) have consistently demonstrated that children from rural and remote Australia, particularly Aboriginal children, have high levels of developmental vulnerability across all five domains: physical, social, emotional, language and culture. In 2018, 54.5% of children in the Fitzroy Valley had developmental vulnerability in 1 domain and 27.3% in 2 domains according to the AEDC. This provides a stark contrast to Australia-wide data, with rates of 21.7 and 11.0% respectively, and indicates the disparity in need of remotely-dwelling Australian children [11]. Wait times for developmental services are long with improvements over recent years (18 months in 2010 to 4 months in 2013) [12]. In 2012, there were 3.6 doctors per 100,000 population in Australia, which trumped the Organisation for Economic Co-operation and Development (OECD) average of 3.3. Unfortunately, the report on Australia’s Future Health Workforce clearly stipulates this does not account for the geographical distribution of doctors, and rural and remote Australia is poorly serviced [13]. Isolation, a measure of remoteness, has a demonstrated detrimental impact on the number of contacts children living in those locations have with doctors [14]. In addition, if their primary carer is Aboriginal they are more likely to be seen by a nurse or Aboriginal Health Worker (AHW) than a doctor [14]*.*

Approximately 20% of the children in the Lililwan cohort (born in 2002 or 2003) were diagnosed with FASD by a multidisciplinary clinical team assessment by 2012 [5, 6, 15]. Many demonstrated chronic and complex needs. Hospital admissions with infections, dental, ear and skin disease, injury and growth faltering were common [4]. Our preliminary data (unpublished) also indicate high rates of emergency department presentations and frequent problems with learning and development [16–18]. Following the Lililwan study, over 400 referrals were made to local health services for 108 children, representing nearly four referrals per child. Nearly half of the children required referral to ear, nose and throat or audiology services; one third had severe dental caries, one third were referred to paediatric or allied health services and 40% to child mental health services (E Elliott personal communication). The burden of complex and chronic health needs documented in the Lililwan Project raised community concerns that child health services were inadequate the very remote Fitzroy Valley. This study was requested by Aboriginal leaders in response to the health service needs identified in the Lililwan Project (2010–2012).

According to the World Health Organisation, the functionality of a health system can be measured by six key factors: health service delivery, health workforce, health information systems, access to essential medicines, health systems financing, and leadership and governance [19]. We utilised the WHO definition of health system functionality as a conceptual framework to examine health service delivery. Other theoretical frameworks informed our study, such as the Penchansky and Thomas theory of access, which describes how the effectiveness of a service depends on optimisation of accessibility; availability; acceptability; affordability; and adequacy in service design, implementation and evaluation [20]. Subsequent modifications by other authors, to the Penchansky and Thomas Theory of Access also incorporate awareness of services as a core domain [21]. Although the core components of this theoretical framework were considered throughout our data collection and analyses, we were unable to address all aspects of their theory because of the limited available data. Specifically, we found no comprehensive overview of all the child health services operating in the Fitzroy Valley in either the published or grey literature and this remains the case.

Our primary objective was to identify and map child health services in the Fitzroy Valley in 2013 to provide a snapshot of the services available to respond to the health needs identified in the Lililwan project (2010–2012) and identify their limitations and barriers to service delivery and access. Data collected included from services involving paediatric specialist, medical officer, allied health, child and adolescent mental health, hospital-based, and emergency services. We also aimed to identify barriers to effective services in this very remote community context.

## Methods

### Identification and description of child health services

In 2014, we approached the regional department of health, child health workers, and searched health department websites to identify child health services operating in the Fitzroy Valley in 2013. We used a semi-structured interview with service providers to confirm the role of services, staffing levels and clinic schedules for 2013. Providers were asked how the referral system worked, what barriers were perceived to affect service provision in remote locations, and how child health services might be made more effective and client-focused. They were asked to clarify the number of full-time equivalent (FTE) staff and the proportion of patient contact time versus travel time, which was not always apparent in schedules. Interviews were conducted with four service managers and 13 practitioners from 17 services including: six from the Western Australian Country Health Services (WACHS); six from the Kimberley Population Health Unit (KPHU); two from Boab Health; two from the Department of Education; and one from the Royal Flying Doctor Service (RFDS). Interviewees included nurses, allied health professionals, specialists, midwives, psychologists, health service administrators and their managers.

We employed qualitative data analysis to gain understanding of the health services available to meet the needs of children in the Lililwan cohort in the region at the time. For this study, we analysed the qualitative data as we engaged in data collection [22]. What we ascertained informed our future interviews and questions.

A content analytic approach was applied to our interview data [22]. We also utilised a narrative analytic approach for data which we obtained from a variety of sources including field notes and documents provided to us during interviews or found on the internet. For this research, our approach was more focused than some qualitative research projects. We were seeking to understand health services, as well as seeking answers to particular questions which we had developed a priorie.

During analysis we categorised the data, indexing the data by our a priorie research questions. The analysis was explanatory and was guided by the research questions. Through our analyses we identified patterns and made connections. We then summarised key themes/ideas from our qualitative findings regarding health services available in the Fitzroy Valley for children with FASD and other developmental, behavioural and mental health needs.

### Ethics

This project was approved by the Western Australian Aboriginal Health Ethics Committee (Approval number 344–04.2011) and the Western Australian Country Health Service Research and Ethics Committee (Approval number 2013:18).

## Results

We could not identify any existing document that provides a comprehensive overview of available services in this region.

### Service providers

**Fig. 2:**
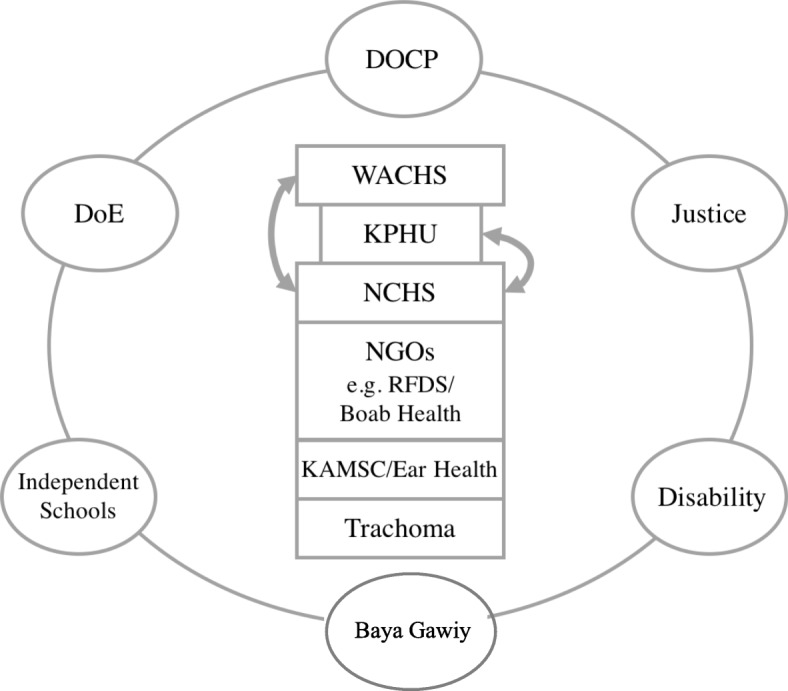
Illustration of the health, education and support services in the Fitzroy Valley and their inter-relationships. Abbreviations: DOCP: Department of Child Protection; DoE: Department of Education; WACHS: WA Country Health Service; KPHU: Kimberly Public Health Unit; NCHS: Nidilingarri Cultural Health Service; NGOs: Non-government organisations; RFDS: Royal Flying Doctors Service; KAMSC: Kimberley Aboriginal Medical Service Council

Using information from multiple sources we identified the following services (Fig. 2):

1. Services provided by WACHS included: paediatric and other specialist services, a paediatric nurse practitioner, acute hospital-based care, emergency evacuations, the Child and Adolescent Mental Health service (CAMH), and a dental service based in Fitzroy Crossing.
2. Services provided by the KPHU included: primary health care, child and maternal health and Allied Health clinics. KPHU is part of WACHS but in many ways, is functionally separate.
3. Services provided by the Aboriginal Medical Service. In the case of the Fitzroy Valley Nindilingarri Cultural Health Services (NCHS) [23] oversees, advises and co-ordinates all government-run health services operating in the Fitzroy Valley via a Memorandum of Understanding with the WACHS and KPHU, effective since 2000. NCHS is the only Aboriginal Medical Service in the Kimberley that does *not* offer a clinical service. Rather, by collaborating with government agencies, it aims to ensure that services are culturally relevant and safe. NCHS does deliver a wide variety of health promotion and disease prevention programs.
4. Services delivered by independent non-government (NGOs) organisations. The RFDS provides emergency evacuations and runs general practitioner (GP) and nursing clinics in certain communities. Boab Health Care [24] is a not-for-profit primary health care organisation that provides services across the Kimberley in mental health, allied health and a range of health promotion programs. Apart from dietetic services, their client base is adults. The Kimberley Aboriginal Medical Services Council (KAMSC) employs an Ear Health Co-coordinator.
5. Other services - An annual trachoma screening and treatment program funded by the Federal Government operates in schools throughout the Kimberley. A program coordinator manages the program and trains local community health nurses in diagnosis and treatment [25].

Schedules of visiting specialists, the paediatric nurse practitioner, Allied Health and Child and Adolescent Mental Health Service teams and others to the Fitzroy Valley are shown in Table 1.

**Table 1:** Paediatric Outreach services to Fitzroy Valley and staffing

Service	Agency	Service base	Regularity of service	FTE staff
Paediatrics	WACHS	Broome	Monthly visit	.2
Allied Health	KPHU	Derby	Weekly visit of 1–3 days	3
Community Health child health nurse	KPHU	Fitzroy Crossing	Full time	1
School health nurse	KPHU	Fitzroy Crossing	Full time	1
Community Health remote area nurses – clinicsat Bayulu, Wangkatjungka and Noonkanbah	KPHU	Fitzroy Crossing	Full time	6
RFDS – 2 x Primary Health Care Nurseand GP Remote clinics	RFDS	Derby	Fixed schedule between weekly andtri-monthly depending on size ofcommunity	2 x .5
CAMHS	WACHS	Broome	One week per fortnight	1
School Psychologists × 2	Department of Education	Fitzroy Crossing and Derby	Full time	2

CAMHS: Child and Adolescent Mental Health Service; KPHU: Kimberley Population Health Unit; RFDS: Royal Flying Doctor Service; WACHS: Western Australian Country Health Services

**Table 2:** Paediatric and child health services in the Fitzroy Valley 2013: by location

*Health professional*	*Visiting schedule*
Fitzroy Crossing (total population approx. 3500)	
Specialist – Paediatrician	Monthly (Mon/Tues/Wed of the week)
Specialist – Paediatric cardiologist	6 monthly
Specialist – Ear/Nose/Throat (not child specific)	4 times yearly
Paediatric nurse practitioner	1 week per month (with paediatrician)
Allied Health team (OT/ST/PT)	Twice monthly (6 days per month)
WA Country Health Service dietician	1 day per month
Child and Adolescent Mental Health Service	Week on/week off (no specific days for visiting remotes)
Department of Education psychologist (4 schools in the Valley including Fitzroy Crossing)	Full time
Department of Education psychologist (4 Independent community schools, outside of Fitzroy Crossing)	Full time
Community health nurse	Full time
School nurse	Full time
Large Remote Communities (total population > 150)	
Noonkanbah (pop.315; 135 km*)	
Specialist – Paediatrician	12 times/ year (Tuesdays)
ENT	Will visit but only if sufficient patients
Allied Health team	1 day/month (Mondays)
Boab Health dietician	1 day/month
Remote clinic	4 × 5 h days per week
VRFDS clinic – GP plus RFDS primary health care nurse CAMHS worker	Every second Wednesday Monthly
Bayulu (pop. 271; 20 km*)	
Specialist – Paediatrician	6 times/year (Thursdays)
Allied Health team	Visit from Fitzroy Crossing – on demand
Remote clinic	4 × 6 h days per month
Boab Health dietician	1 day per month
Wankgatjungka (pop. 169; 140 km*)	
Specialist- Paediatrician	6 times/year (Thursdays – alternatemonths with Bayulu)
Allied Health team	12 times/year (Wednesdays)
Remote clinic	4 × 5 h days per week
Boab Health dietician	1 day per month
CAMHS worker	Monthly
Small Remote Communities (total population < 100)	
Yakanarra (pop. 100; 150 km*)	
Allied Health team	3 times/year
RFDS clinic – GP plus RFDS nurse	Weekly
Boab Health dietician	1 day/month if seat available on RFDS plane
Koorabye (pop. 64; 100 km*)	
RFDS clinic – GP plus RFDS nurse	Twice monthly
Boab Health dietician	1 day/month if seat available on RFDS plane
Djugerari (pop. 59; 125 km*	
Allied Health team	3 times/ year
RFDS clinic – GP plus RFDS nurse	Weekly
Boab Health dietician	1 day/month if seat available on RFDS plane
Kadjina (pop. 39; 210 km*)	
Allied Health team	3 times/ year
RFDS clinic – GP plus RFDS nurse	1 day/month
Boab Health dietician	1 day/month if seat available on RFDS plane

Population derived from Morphy F [9]

*Approximate distance from Fitzroy Crossing

Other agencies act collaboratively at a local level to address child health and wellbeing in the Fitzroy Valley (Fig. 2). These include the Department of Child Protection, the Department of Education (a nurse and a psychologist are employed at the Fitzroy Crossing District High School for example, and another psychologist oversees all four Independent Community Schools in the Kimberley) and Baya Gawiy, the Children and Family Centre in Fitzroy Crossing, established in 2013. Marninwarntikura Women’s Resource Centre [26] manages Baya Gawiy, a domestic violence shelter, and the Marulu Unit. This unit was established with a full-time youth worker to respond to findings of the Lililwan project, to support children and families living with FASD and coordinate involvement by agencies in the interests of families and children. The Department of Justice, in its support of juvenile offenders is also part of this picture. The Disability Services Commission WA has an office in Fitzroy Crossing and provides support to eligible children and families. Figure 2 illustrates the health services in the Fitzroy Valley.

### Fitzroy crossing hospital

Fitzroy Crossing hospital, run by WACHS, is the hub of the health system in the Fitzroy Valley. On average the hospital is staffed by eight nurses (from a pool of 19) and three medical officers. It has a Level 3 paediatric facility [27] with an emergency department and 12 inpatient beds (2 designated for children) staffed by a Medical Officer. Two inpatient beds are designated for outpatients requiring dialysis. Doctors usually have limited post-graduate training in paediatrics. On most days one of the three doctors will be away working in a community clinic. The medical positions are funded but not always filled. In 2013 only one of the doctors resided permanently in Fitzroy Crossing and the others travelled from Derby or Broome. The hospital employs three Aboriginal Liaison Officers whose primary role is to locate, and provide a transport service for, patients to attend medical appointments.

According to the nature and severity of their illness and the availability of beds and staff, children may be transferred to the regional hospital in Broome for Level 5 paediatric care (400 km) [27], where there is an 8 bed paediatric ward staffed by a resident medical officer, a paediatric registrar, and an on-site consultant paediatrician. Two paediatric registrars undertake 6 or 12-month rotations to Broome from the Princess Margaret Hospital for Children in Perth. Acutely ill children may also be transferred from Fitzroy Crossing to the Level 3 paediatric care hospital in Derby (260 km) [27], which does not have a paediatrician on-site, or to the Level 6 tertiary hospital (Princess Margaret Hospital for Children) in Perth (2500 km) [28] .

Few people in the Fitzroy Valley own cars and there is no public transport. The mode of patient transfer is dictated by the severity of the patient’s condition and the destination and includes evacuation by the RDFS and road ambulance to Derby or Broome. There are two road ambulances at the Fitzroy Crossing Hospital, however patients from remote communities often drive to meet the ambulance half-way, to halve the time the ambulance is out of reach. Patients may also take the daily (except for Saturday) Greyhound bus from Fitzroy Crossing to Derby or Broome if their condition allows, or use the ‘linen run’, which transports hospital laundry to Derby. During our audit the Greyhound bus departed Fitzroy Crossing at 1:00 am, arrived into Derby at 4:00 am ($62 AUD) and into Broome ($92 AUD) at 6:40 am.

### Services in Fitzroy Crossing

The visiting schedule for all services for Fitzroy Crossing in 2013 is shown in Table 1. In 2013 there were 6 individual paediatricians comprising three FTE consultant positions. These consultants serviced the entire Kimberley, a vast geographical area incorporating hundreds of remote communities, and provide outreach services from Broome to Derby, Fitzroy Crossing, Kununurra, Halls Creek and communities on the Gibb River Road and around Balgo (Fig. 1). When possible, individual paediatricians provide a service to specific communities on a regular basis to increase continuity of care. One paediatrician, the only one with a full time equivalent position, resided ‘locally’ in Broome, which is approximately 400 km from Fitzroy Crossing. All the others flew in from Perth (1) or interstate: NSW (1), Victoria (2) and NT (1) and work between 0.1 and 0.5 FTE. In 2013, two of the paediatricians attended clinics in Fitzroy Crossing and surrounding communities, accompanied by one of the two paediatric registrars. Locums are sometimes employed to cover consultant and registrar leave and illness.

### Services in outlying communities

Outside Fitzroy Crossing, the three largest communities in the Fitzroy Valley are Noonkanbah, Bayulu and Wangkatjungka (Table 2). Bayulu is closest to Fitzroy Crossing (10 km south). Wangkatjungka (130 km south east; 100 km bitumen, 30 km unsealed) and Noonkanbah (165 km west; 100 km bitumen) can only be accessed via significant sections of unsealed road, which can become impassable in the wet season (December to April). Each community has its own remote health clinic, serviced by remote area nurses and nurse generalists employed by the Community Health service, administered by the KPHU. They operate in isolated and demanding environments. Their portfolio, according to the remote clinic manager based in Fitzroy Crossing, includes:

*“ … sexual health, antenatal and postnatal child health, immunization, rheumatic heart disease, adult health, aged care, chronic disease pathways including for diabetes, cardiac and respiratory conditions, communicable disease, wound care, primary health care and all acute presentations to remote clinics”.*

**Fig. 3:**
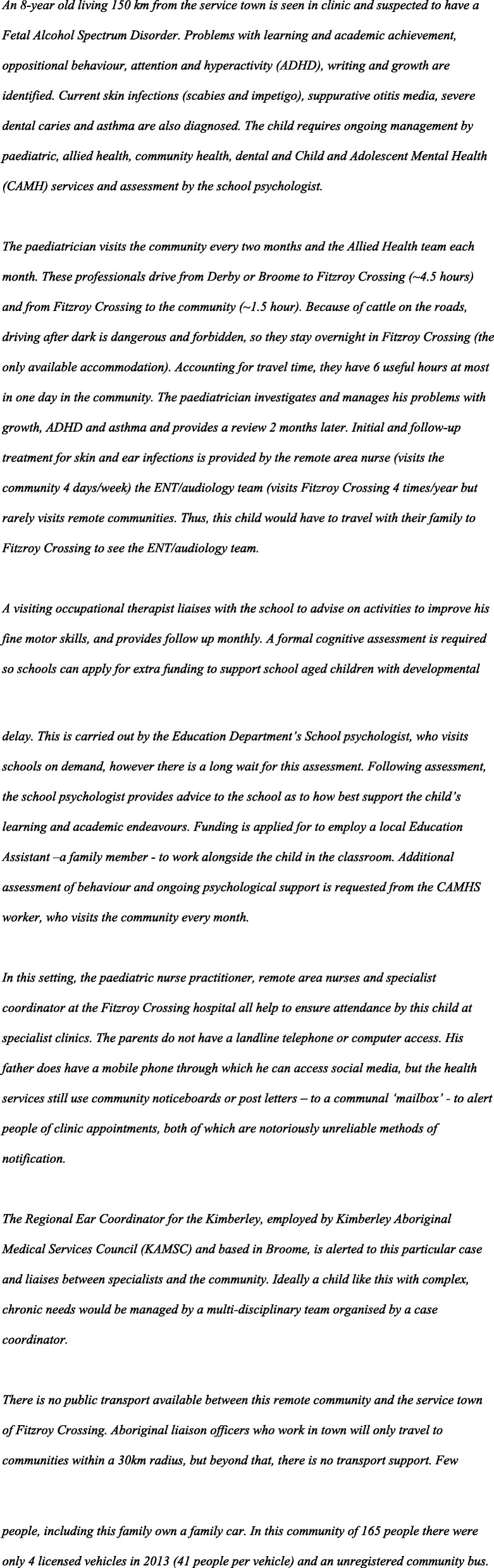
Fictitious case illustrating challenges in accessing services

Paediatricians and allied health professionals visit the three remote clinics every 8 weeks and the RFDS runs a fortnightly GP clinic in Noonkanbah with a district medical officer from Fitzroy Crossing (Table 1). To demonstrate the difficulties in accessing services in outlying communities, a fictitious, but representative, case with chronic and complex health and developmental needs is illustrated in Fig. 3.

### Services in smaller, more remote communities

People in the 42 remaining smaller communities (which range from approximately 40 to 300 people) [10] receive health care from clinics at the three larger communities or hospital-based clinics in Fitzroy Crossing. The Allied Health team and RFDS visit four of these communities (Yakanarra, Kadjina, Djugerari and Koorabye) periodically, ranging from weekly to monthly (Table 1).

### Paediatric services

A paediatrician visited the Fitzroy Valley every two months and the Allied Health team visits each month. These professionals drive from Derby or Broome to Fitzroy Crossing (~ 4.5 h) and from Fitzroy Crossing to the outlying, smaller communities, on average about 1 h from Fitzroy Crossing. Due to unfenced cattle on the roads, driving after dark is dangerous and not recommended, so staff stay overnight in Fitzroy Crossing (the only available accommodation). Accounting for travel time, they have 6 h available to spend in the community. The paediatrician investigates and manages health problems, for example: growth, attention deficit hyperactivity disorder, and asthma and provides a review 2 months later. Initial treatment for skin and ear infections is provided with follow-up by the remote area nurse (visits the community 4 days/week) and the ENT/audiology team (visits Fitzroy Crossing 4 times/year but does not visit remote communities with the occasional exception of Noonkanbah via RFDS if there are sufficient referrals). A child who needs ENT/Audiology review must travel with their family to Fitzroy Crossing to see the ENT/audiology team.

#### One clinician reported to us

*“ … physically getting the kids to the clinic to see me is the greatest influence on how many kids I have seen so far, school age kids in particular. Very tricky to work around school hours, I am typically gone by 2-2.30 [to complete the return drive back to Broome] so often miss the afterschool window and have rung schools before but cannot see kids without parental consent/presence and mum might be at work or uncontactable. Availability of Aboriginal health workers is crucial – and I notice a significant reduction in numbers.”*

A visiting occupational therapist liaises with the school to advise on activities, such as those to improve fine motor skills and provides monthly follow-up. A formal cognitive assessment is required before schools can apply for extra funding to support school aged children with developmental delay. This may be carried out by the Education Department’s School psychologist, who visits remote schools upon request. However, there is often a long wait for these assessments. Following assessment, the school psychologist provides advice to the school as to how best support the child’s learning. Funding may be justified for employing a local Education Assistant – often a family member - to work alongside the child in the classroom. Additional assessment of behaviours and ongoing psychological support can be provided by the Child and Adolescent Mental Health Service (CAMHS) worker, who visits the community every month.

Based on Lililwan data up to 20% of children in the Fitzroy Valley have FASD with complex, chronic needs as in the child described in Fig. 3. These children would optimally be managed by a multi-disciplinary team with a case coordinator, but such services are lacking. In the Fitzroy Valley, the paediatric nurse practitioner, remote area nurses and the specialist coordinator at the Fitzroy Crossing hospital all help to ensure attendance by children with complex, chronic needs at specialist paediatric clinics in Fitzroy Crossing. There is a Regional Ear Coordinator for the Kimberley, employed by KAMSC and based in Broome, who can be alerted to particular children’s needs and liaise between specialists and communities.

There is no public transport available between remote communities and the service town of Fitzroy Crossing. Aboriginal liaison officers work in town and travel to communities within a 30 km radius to provide transport, but beyond that, there is no transport support. Few people own a family car. In 2013, in one community of 165 people there were 4 licensed vehicles (41 people per vehicle) and an unregistered community bus. Few houses have landline telephones or computers. Many people now have mobile phones through which they access the internet and social media, but health services still use community noticeboards and post letters – to a communal ‘mailbox’ - to alert people of clinic appointments, notoriously unreliable methods of notification.

### Allied health

All allied health workers in 2013 were based in Derby, and travelled from there (260 km, 40 min by air) to Fitzroy Crossing. Two allied health teams operated in the Fitzroy Valley. These teams consist of an occupational therapist, a physiotherapist, a speech therapist and an Indigenous therapy assistant. Their visiting schedule for the Valley over four weeks is 3 days/2 days/4 days/2 days, or 11 days per month. In the weeks when they visit for three or four days, the team drives out from Derby. On the 2-day weeks - every fortnight - they fly with the RFDS to remote communities or charter a flight. When in town for three or four days, they drive out to remote communities from Fitzroy Crossing (see Table 1). This team also services the town and communities of Derby and the communities of the Gibb River Road and Looma.

Most of the occupational and speech therapist caseload is children, and the team liaises closely with all schools in the Fitzroy Valley and with Baya Gawiy, the Children and Family Centre in Fitzroy Crossing. In late 2012, funding was secured for a 1.0 FTE occupational therapist to work exclusively with children in the Fitzroy Valley. Referrals to Allied Health can be made by paediatricians, remote clinic nurses, teachers and school nurses. Visiting therapists must prioritise the most acute problems, at times displacing children with less urgent problems who are on their waiting list. A monthly meeting is held between the allied health team and the paediatrician in Fitzroy Crossing, with a focus on children with developmental delays and other complex needs.

### Child and adolescent mental health service (CAMHS) and school psychologists

There was one FTE child and adolescent mental health worker (a clinical psychologist, nurse or social worker can hold the position) based in Fitzroy Crossing in 2013. Due to a lack of accommodation, this person spent every second week in Broome dealing with administration and referrals from the week spent in Fitzroy Crossing. There was also one FTE Indigenous mental health worker who works with families (adults and children together), and acts in an advisory role to the other worker. Most referrals to CAMHS come from health services and government agencies, including the Department of Child Protection, Department of Education (school psychologists) and Department of Justice (Youth Justice worker). One day each fortnight is allocated to providing services to more remote communities if there is a child requiring services. Thus, the three larger communities may see this worker every two to 3 months if there is a child in ongoing care. Only in extreme cases do mental health patients get evacuated out of Fitzroy Crossing (to Perth), because the experience of being removed from familiar surroundings can be extremely traumatic. Although available for consultations via teleconference, the child psychiatrist visits the Kimberley for only 1 week per year, leaving mental health workers to take considerable clinical responsibility.

There are two school psychologists in the Fitzroy Valley. One is based at the Fitzroy Crossing High School and covers the Department of Education’s other four remote community schools. The other is based in Derby and covers the four Independent (non-government) community schools in Fitzroy Valley. Their predominant role is to guide teachers and families in managing challenging behaviour and academic difficulties in the school setting. They also conduct cognitive assessments, which may be used by the school to apply for extra funding to support students with cognitive deficits and learning problems. Historically, these assessments have not been shared with service providers outside of the Education Department including the Health Department.

### Paediatric nurse practitioner

A paediatric nurse practitioner (PNP) position was initiated in April 2013 in response to concerns that there was inadequate coordination of paediatric clinic lists, resulting in few referrals and a limited focus on children most in need of specialist care. The number of children on the paediatric list almost trebled with this appointment, suggesting that there was underservicing of children previously.

The PNP’s role is to coordinate services for children with referrals to paediatric practitioners, numbering over 300 in 2013. This requires liaison with remote area nurses (RANs) in the three remote clinics, and with primary health care nurses for the RFDS clinics to organise referral lists prior to the paediatricians’ visits to outlying communities. Children with complex medical conditions, including growth faltering, are seen by the PNP in the week prior to clinics. The PNP manages a case-load of children with chronic illness in partnership with the district medical officer and paediatrician, and liaises with Aboriginal health workers, where available, who communicate with families to inform them of impending visits. When Aboriginal health workers are not available, families are notified by whatever means available (e.g. door knocking, Facebook, or telephone if available). Ongoing referrals are also managed by the PNP, including those to the Princess Margaret Hospital’s ambulatory care service in Perth. In 2013, the PNP began to develop ‘chronic disease plans’ for specific children, which were made available to all practitioners via the Communicare™ patient database. The PNP plays a crucial role in communicating with families about details of their medications, identifying the best family members to take on supervisory responsibilities relating to medication and diet, and liaising with other agencies where necessary.

There is a ‘Specialist Booking Co-ordinator’ responsible for generating patient lists and visit dates for all visiting specialists, excluding paediatricians. Paediatrician visits are organised by the Population Health Child Health Nurse. This role has previously had more staff turnover than the specialist booking co-ordinator. With each change of staff, time to fully grasp the context and requirements of the job is required, as well as re-establishment of systematic approaches to the role. This disjunction could have contributed to issues related to capturing the children that needed to be seen. Providing administration assistants or centralisation of this role within the hospital could improve outreach capacity of Child Health Nurses, along with AHW, by allowing them to redirect their time expenditure towards seeing children rather than grappling with technology and databases, ultimately improving functionality of this role and capitalising on the systems already in place, making a more robust and consistent system to capture the children who needed to be seen (personal communication, Ruth Kinninburgh-White).

### Community health services

The Community Health service, which is part of the KPHU, employs one child health nurse based in Fitzroy Crossing. There is no requirement for training in child health or Aboriginal health, however the nurse oversees the immunisation program throughout the Valley and runs the Under-5 s program, which aims to review all children on a regular basis. This nurse is also responsible for monitoring babies deemed ‘at risk’, that is, pre-term, underweight or suffering from congenital anomalies. The Community Health midwife estimates that of the 76 babies born in 2012–13, ten (13.2%) were in this category. The child health nurse also assists with coordinating paediatric clinics in Fitzroy Crossing, managing referrals from Fitzroy Crossing-based medical officers and the School health nurse. The School Health nurse, also employed by KPHU, is responsible for developmental and general health checks for all school aged children and the child health nurse is responsible for these same checks in children aged five and under.

### Dietician services

WACHS and Boab Health Service both provide paediatric dietician services to the Fitzroy Valley. WACHS serves residents of Fitzroy Crossing town and communities of Kadjina and Yakanarra, provided a referral has been made and there is a seat available on the RFDS flight. The WACHs dietician visits Fitzroy Crossing, using the RFDS flight from Derby, once per month, holds her clinic at the hospital and is assisted by the Aboriginal Liaison Officers in finding her clients. The Boab Health dietician, who has both paediatric and adult clients, visits outlying communities only, namely Bayulu, Wangkatjungka and Noonkanbah. She spends 1 week per month in the Fitzroy Valley (10 visits per year) and also visits the smaller communities of Koorabye and Djugerari if there is a seat available on the RFDS flight.

### Cultural brokers: aboriginal health workers

All health service providers interviewed emphasized the importance of Aboriginal Health Workers (AHW) or liaison officers/cultural brokers and noted that the effectiveness of services was diminished by the absence of AHWs. Currently, there are only two AHW in the entire Fitzroy Valley, three Aboriginal liaison officers employed by the hospital, and one Aboriginal therapy assistant who travels with the Allied Health team from Derby.

## Discussion

The delivery of health services in remote settings is complex. This study highlights health workforce shortages in the Fitzroy Valley, in particular for medical staff and community nurses, with a severe shortage (only 1/3 of the required number) of AHW [29]. Barriers to delivering an effective service, identified in interviews with staff across agencies, were consistent with those documented in the literature. Unsealed roads and climatic factors such as the tropical wet season limit access to health service facilities for people living in remote communities and by nurses and doctors to those communities. Limited accommodation is an invisible barrier to health care delivery and often means that health staff must fly or drive in/out. This is expensive, time-consuming and may result in inconsistent health care from a workforce not well known to or culturally informed about the community.

The challenges we identified in health service delivery in remote settings is reflected in the few reports available that describe the institutional landscape of health services in the Kimberley [30, 31]. Lewis (2013) detailed challenges including: population growth of 2% per year with a predominance of young people (25% of the population is aged 0–9 years) in the Kimberley Aboriginal community; high rates and burden of ill-health experienced by the community compared to both Indigenous and non-Indigenous populations elsewhere in Western Australian (e.g. infant mortality rates 1.2 times higher than WA Indigenous populations and 4 times higher than in the total state population from 1998 to 2007); high costs of service delivery in the Kimberley compared to metropolitan areas; difficulties with recruitment and retention of staff due to national shortages of those with appropriate skill sets; and fundamental obstacles due to structural issues such as lack of housing for staff in remote areas [32]. Atkinson et al. (1999) described deficiencies in funding for Aboriginal health care services across Australia and the skewed distribution of expenditure on hospital services for Aboriginal compared to non-Aboriginal people, indicating poor use of primary health care services and resultant late presentations [29].

The role of child health nurses, Aboriginal liaison officers and others in assisting families to navigate the health system is crucial. While health practitioners ‘on the ground’ strive to work together to achieve the best possible outcomes for their paediatric patients, the institutional landscape in which they operate is a hindrance. This includes the lack of consistent and up-to-date documentation or a ‘one-stop-shop’ source of information regarding health service provision in the region. Difficulty recruiting and retaining health professionals is a significant and well-documented issue [3, 29, 32, 33]. The dearth of Aboriginal health workers (AHWs) documented in our study (two per 3100 population) is not a new observation in the Kimberley, recruitment of AHW having been identified as a top priority 20 years ago in the 1999 Kimberley Regional Aboriginal Health Plan [29]. There are no staff to population ratios recommended for WA however, in Central Australia, they recommend one AHW per 100 Aboriginal population, one community nurse per 250 and one doctor per 600 are likely applicable in the Kimberley [30].

The lack of AHW was deemed problematic by all service providers. AHW know local families, communities and the context in which health services are delivered, speak local languages and understand cultural protocols. This knowledge is essential to assist non-Aboriginal clinicians to encourage Aboriginal people to attend clinics. Trained AHW also have a vital role in two-way interpreting between health practitioner and patient, providing access into communities, and an explanation of reasons for non-attendance and non-compliance [34].

AHW are crucial for child health services in liaising with parents or extended family members caring for children [35] and in remote communities their roles include supervision of immunisation, medications, child health surveillance and recognition and referral of the acutely ill child. Although training programs for AHWs are well subscribed [29], few Aboriginal people are retained in these positions. Reasons for this include inadequate supervision and support at a community level, and a lack of career path. In addition, the training is conducted in Derby or Broome, which requires the AHW to separate from their family and community for the duration of the training. Some concerted focus at a regional level to improve the appeal of the role of AHW is required. Offering AHW courses in Fitzroy Crossing could potentially improve recruitment and retention.

High staff turnover results in a lack of continuity of local and cultural knowledge – especially of children and their extended families. This results in a constant need for Aboriginal people to build new relationships, which might impact on their willingness to engage with the health system at all. It leads to confusion amongst service providers as to who to contact or where to refer patients and is expensive. A regularly updated, online calendar of health services including visiting schedules, names and contact details, could have contributed to the delivery of more coordinated services in 2013. Such a calendar was developed by Patches, a paediatric outreach health and education service, and trialled in 2014 on their website but required maintenance [36]. Although the calendar exists on the Patches website, the calendar is updated infrequently. The last calendar entry for Fitzroy Crossing was June 2019, prior to that in August 2016, and for Broome was August 2017.

Providing additional housing for health workers – which requires negotiation with traditional land owners and planning involving many agencies in Fitzroy Crossing – would enable health professionals to live in Fitzroy Crossing and build relationships with colleagues working in the area. The fly-in fly-out approach has major limitations including restricting the time available to provide clinical care and professional support.

Challenges exist for families in knowing when health professionals will be in town and balancing clinic attendance with other commitments. A particular challenge for specialists and allied health teams in remote clinic contexts is in notifying families of patients of impending visits and locating children on the day. Children represent a particular challenge due to limitations surrounding inability to review children during school hours, resulting in a small window of time after school.

Conversely, as we identified, there is a complex and variable schedule by which many paediatric service providers visit Fitzroy Valley communities. This information about who is visiting and when may be confusing. It is not uncommon to find clinic appointment letters written in English scattered unread in communities. Technology including access to mobile phones and messenger applications may assist in the future.

Some practitioners observed that an opportunistic approach to assessment and treatment can work when parents bring children to the clinic for other matters – but in these instances, the Communicare™ online database containing medical records and histories for patients may not be available, making treatment uncoordinated and possibly ineffective or even risky. The paediatric nurse practitioner said that she prints out records for all referrals, and physically takes them to remote clinics, in case the online record system is not available.

Although it was difficult to measure, evidence for chronic under-resourcing can be found in the approach that services are forced to take in the Fitzroy Valley, namely a triage approach. Using this approach, the most acute patients are seen on any given day or at any given clinic, while those with chronic illness, particularly children who are difficult to access, slip down the priority list or become lost altogether. The conflict between the demands for attention to acute matters, versus the need for primary or ongoing health care, has been well-documented elsewhere in remote Australia. Gruen and Bailie noted that “according to the way in which (specialist) outreach is conducted and the service is organised, it can either support primary care or it can hinder primary care and, as a result, reduce its own effectiveness [37].”

Resources are needed for provision of quality, ongoing primary health care that is quarantined from acute demands. The intense focus on responding to acute care needs is apparent in the design of clinics recently built at considerable expense at Bayulu, Wangkatjungka and Noonkanbah. There is no space within these clinics for the provision of primary health care, nor any desk, office space or computer access for AHWs.

Studies from the Northern Territory have identified poor co-ordination and poor communication and linkages between health services as barriers to accessing health care, especially for people with chronic, complex disorders [31, 37, 38]. Our experience in the Lililwan study and reports from health service providers suggest similar problems in the Fitzroy Valley to the detriment of children and families. For example, our data showed a physiotherapist, paediatrician and occupational therapist may visit a community on three subsequent weeks to see the same child but be unaware of each other’s findings. Across WA there is a recognized lack of integration and coordination between services, as well as service inconsistencies, primarily due to a lack of an overarching framework or model to describe child health and developmental service delivery [12]. Primary specialist health care must be integrated, ideally with dedicated coordinators across services [39]. Our efforts to map services in the Fitzroy Valley for children in Lililwan cohort with chronic complex neurodevelopmental needs illustrate the lack of integration and provides a baseline for development of future, better coordinated services that improve access, minimize early life health inequities and optimize child health and development [39, 40].

Other barriers to health care include poverty and other social factors [41–44] and uneven distribution of health professionals [27, 45, 46]. Models of best practice for remote settings are client-focused services delivered by a multi-disciplinary team that can demonstrate consistency and commitment over time [37, 47–60]. A multi-disciplinary team approach, such as that used in the Lililwan study, makes particular sense when populations have complex health needs [23, 24, 34, 38, 55]. This approach facilitates efficient, comprehensive assessment, diagnosis and development of management plans for children with particularly complex needs. It promotes cross-disciplinary communication, limits duplication of effort and provides health professionals (many of whom are junior) with a professional support and supervisory network, likely improving staff confidence and retention.

The paediatric nurse practitioner appointed to the Fitzroy Valley in 2013 was a crucial structural element in the functionality of the system overall. This nurse acted as an intermediary between complex health systems and remote Aboriginal communities and had a coordinating role. Ideally such a position would be supported by AHWs to facilitate cultural competence and overcome language barriers.

In 2013, Patches Paediatrics, a private multidisciplinary child health enterprise, commenced work with Nindilingarri Cultural Health Service (Fitzroy Crossing) and WA government-funded health services to coordinate outreach schedules and establish a family-centred approach to addressing complex neurodevelopmental issues [26]. Patches received government, philanthropic and research funding to deliver multidisciplinary clinics that improved the coordination, efficiency and effectiveness of the current health and education services for children with complex needs in the Fitzroy Valley [26, 36]. Another initiative for the Fitzroy Valley was the Kimberley Dental Team, a not-for-profit organisation founded in 2009 that broadened its scope from the East Kimberley to the whole Kimberley from 2014 and continues to provides dental services in the region [61]. There is a new ambulance (since 2014) and clinic based at Wangkatjungka (provided by Kurungal Council Inc., June 2019) to transfer patients into Fitzroy Crossing. In 2015, Marninwarntikura partnered with clinician-researchers at the University of Sydney to adapt and introduce the evidence-based Triple P-Positive Parenting Program which has had a positive impact on parent skills and knowledge and wellbeing of children and families in the wider community [62]. Another partnership, which commenced in October 2016 between Royal Far West and Marninwarntikura Women’s Resource Centre under an initial five-year agreement, assists in providing children in the Fitzroy Valley with paediatric, psychiatric and allied health care that is FASD and trauma-informed. This includes in-person and on-going tele-paediatric and allied health service [63]. Progress is being made to improve coordination within health services (via online calendars) [36] and between health services and others such as the Department of Education in terms of sharing information and assessments, however there is still much to be done to streamline and coordinate health care services and child health review and management [64]. Furthermore, the NDIS roll-out will likely shift the landscape for service provision in the Kimberley, however this process will require time to implement and see change.

Moving to the future, there is a need for a formal, prospective audit of child health services in the region, with development of services to fill gaps in consultation with community and integration of services to maximise efficiency and minimise duplicity. Informed by our work with the Lililwan cohort, services should ideally be multi-disciplinary, trauma-informed, capable of acute and chronic complex care, culturally appropriate and inclusive of AHW. Although it was beyond the scope of our study to evaluate existing services, future work should encompass this.

Although the audit was done in 2013 in response to the clinical demand identified in the Lililwan project this should not be seen as a limitation: it remains relevant because demand is ongoing, and services remain uncoordinated and under-resourced. The strengths of the current work are that it was requested by the community and has been used by them to advocate for change. This work provides baseline information to allow comparison with current and future service provision. Personal correspondence with Royal Far West (a Sydney provider of health services in the Fitzroy Valley) in August 2019 indicated that they still refer to the data in this paper as the only clear outline of available services and providers and that no comprehensive overview of current services exists.

This audit highlights complexities in service provision for remote dwelling Australian Aboriginal children living across the 45 communities in the Fitzroy Valley. In particular, these complexities are encapsulated within physical and invisible barriers, health workforce recruitment and turnover, the intricacies of piecemeal funding across multiple levels of government and NGOs, as well as lack of integration and coordination of services and implications on accessibility of health services even if they exist.

## Abbreviations

AEDC: Australian Early Development Census
AHW: Aboriginal Health Worker
CAMHS: Child and Adolescent Mental Health Service;
DOCP: Department of Child Protection
DoE: Department of Education
ENT: Ear, nose and throat
FASD: Fetal Alcohol Spectrum Disorders
FTE: Full-time equivalent
GP: General practitioner.
KAMSC: Kimberley Aboriginal Medical Services Council
KPHU: Kimberley Population Health Unit
NCHS: Nindilingarri Cultural Health Services
NGOs: Non-government organisations
PNP: Paediatric Nurse Practitioner
RAN: Remote Area Nurse
RFDS: Royal Flying Doctor Service
WA: Western Australia
WACHS: Western Australian Country Health Services
WHO: World Health Organisation.
